# Antimicrobial Potential of Endophytic Fungi From *Artemisia argyi* and Bioactive Metabolites From *Diaporthe* sp. AC1

**DOI:** 10.3389/fmicb.2022.908836

**Published:** 2022-06-23

**Authors:** Haiping Gu, Shikai Zhang, Lin Liu, Zhengyou Yang, Fengchun Zhao, Yuan Tian

**Affiliations:** ^1^Key Laboratory for Agriculture Microbiology, Department of Microbiology, College of Life Sciences, Shandong Agricultural University, Taian, China; ^2^College of Life Sciences, Shandong First Medical University and Shandong Academy of Medical Sciences, Taian, China

**Keywords:** *Artemisia argyi*, endophytic fungi, antimicrobial activity, cytotoxic activity, secondary metabolites

## Abstract

Endophytic fungi of medicinal plants are important sources of active natural products. In this study, 26 fungi were isolated from *Artemisia argyi*, which were belonging to eight genera, namely, *Alternaria*, *Fusarium*, *Chaetomium*, *Phoma*, *Diaporthe*, *Trichoderma*, *Gibberella*, and *Colletotrichum*. The antimicrobial activities of all fungal extracts were tested by using the cup-plate method against *Staphylococcus aureus*, *Salmonella enteritidis*, and *Fusarium graminearum*. The results demonstrated that 25 extracts (96%) exhibited inhibitory activity against at least one of the tested pathogenic microorganisms. The strain *Diaporthe* sp. AC1, which showed good antimicrobial activity and high yield of crude extract from fermentation, was selected for the study of secondary metabolites. The crude extract of strain AC1 was purified by silica gel column chromatography, Sephadex LH-20 gel column chromatography, and HPLC, and finally, a new compound phomopsolide G (**1**), together with three known phomopsolides (**2**–**4**) and four other known compounds (**5**–**8**), was obtained. The structures of the compounds were elucidated by NMR and/or HR-MS spectroscopy. Microdilution method and MTT colorimetry were used to determine the bioactivity of the compounds. The study demonstrated that the new compound **1** had moderate antifungal activity against *F. graminearum*, *Fusarium moniliforme*, and *Botrytis cinerea* and weak antibacterial activity against *Staphylococcus aureus.* Compound **1** also showed weak cytotoxicity against HepG2, A549, and MDA-MB-231, with IC_50_ values of 89.91, 107.65, and 53.97 μM. Additionally, other compounds also exhibited antimicrobial and/or cytotoxic activities. The findings provided the basis for searching drug and agricultural lead compounds from *A. argyi*-associated fungi resources.

## Introduction

In modern agriculture, plant pathogen is a main concern because of its negative effect on crop yield and quality, which leads to serious economic losses (Ab Rahman et al., 2018). Therefore, to reduce the harmful effects of multiple phytopathogens, finding antagonistic agents for the development of pesticides is urgently needed. In addition, human health is at constant risk due to the occurrence of different types of communicable diseases (CDs) and non-communicable diseases (NCDs). Antibiotic is an effective means of treating CDs. However, antibiotic resistance has become one of the most serious challenges in the world (Petchiappan and Chatterji, 2017). Drug-resistant infections result in approximately 23 thousand deaths in the United States and 25 thousand deaths in Europe annually, and much higher numbers of deaths in developing countries (Blair et al., 2015). On the contrary, three out of every 10 premature deaths from NCDs are due to cancer (Bray et al., 2021). As estimated by the International Agency for Research on Cancer (IARC), there were 19.3 million new cases of cancer and almost 10.0 million deaths from cancer worldwide in 2020 (Ferlay et al., 2021). The search for new drugs to treat infections and cancers is also urgent.

Natural products, especially that originated from microorganisms, have provided amounts of lead molecules that can be manipulated to produce desirable novel analogs for agricultural and therapeutic use. In the last few decades, endophytic fungi, which reside in plant tissues without showing any disease symptoms, have gained extensive attention due to their ability to produce kinds of secondary metabolites (Manganyi and Ateba, 2020). The natural products derived from fungal endophytes possessed a variety of biological properties, such as antimicrobial (Joo et al., 2021), antiparasitic (Moreno et al., 2011), antioxidant (Mishra et al., 2020), immunosuppressant (Wang et al., 2019), and anticancer activities (Li et al., 2018; Uzma et al., 2018).

*Artemisia argyi* is known as “Aicao” in China. It belongs to the Asteraceae family and is widely distributed in Asia, Europe, and North America (Bora and Sharma, 2011). As a traditional Chinese herbal medicine, *A. argyi* is used to control dysmenorrhea, abdominal pain, and inflammation (Chinese Pharmacopoeia Commission, 2015). Phytochemical analysis revealed that *A. argyi* was rich in essential oils (Abad et al., 2012), flavonoids (Han et al., 2017; Lv et al., 2018), terpenes (Zhang and Lv, 2019; Ming et al., 2021; Zhao et al., 2021), and other secondary metabolites (Lan et al., 2020). Further study indicated that the compounds had promising biological activity, such as antifungal (Wang et al., 2022), antioxidant (Kim et al., 2015), antiproliferative (Ming et al., 2021), anti-tumor (Zhang et al., 2021), anti-inflammatory (Yun et al., 2016), anticoagulant (Lv et al., 2018), and immunoregulatory activities (Zhang et al., 2018).

However, only a few studies on endophytic fungi from *A. argyi* have been reported. From the culture extract of *Trichoderma koningiopsis* QA-3, an endophytic fungus obtained from *A. argyi*, 13 new fungal polyketides were isolated. Some of them displayed antibacterial and antifungal activities (Shi et al., 2017, 2020). Chemical investigation of another *A. argyi*-derived fungus *Trichoderma virens* QA-8 obtained 11 new sesquiterpenes, some of which showed antimicrobial activity (Shi et al., 2019, 2021). A study on the diversity of endophytes in *A. argyi* from different areas of China has revealed that their fungal community structures and diversities were quite different (Wu et al., 2021). It is believed that the exploration of underexploited *A. argyi*-associated endophytic fungi will enrich the resources of endophytes and then lead to the discovery of novel bioactive compounds.

## Materials and Methods

### Pathogens and Media

Pathogenic bacteria, including *Staphylococcus aureus* (ATCC25923), *Escherichia coli* (ATCC8739), *Salmonella enteritidis* (ATCC13076), and *Pseudomonas aeruginosa* (ATCC27853), were purchased from Shanghai Bioresource Collection Center (SHBCC). The media used in this study were potato dextrose agar (PDA) medium (200 g potato, 20 g dextrose, 15 g agar, 1,000 ml deionized water), malt extract (ME) liquid medium (20 g raw malt, 20 g sucrose, 1 g peptone, 1,000 ml deionized water), and Luria-Bertani (LB) medium (10 g tryptone, 5 g yeast extract, 10 g NaCl, 1,000 ml deionized water, pH 7.2).

Pathogenic fungi including *Fusarium graminearum*, *Fusarium moniliforme*, *Botrytis cinerea*, and *Verticillium dahlia* were all separated and identified by our laboratory. *F. graminearum* was isolated from wheat with Fusarium head blight. *F. moniliforme* was isolated from pepper with root rot. *B. cinerea* and *V. dahlia* were isolated from tobacco with gray mold and verticillium wilt, respectively. The identification of the four plant pathogenic fungi was based on the observation of microscopic morphology (Supplementary Figure 1) and phylogenetic analysis (Supplementary Figure 2).

### Material Collection and Fungi Isolation

The healthy *A. argyi* used in this study was collected from the Shandong Agricultural University, Tai’an, Shandong Province, China. Endophytic fungi were isolated according to the methods detailed previously (Liu et al., 2019) with some modifications. First, the tissues (leaves, stems, and roots) were rinsed with clean water to remove dust and then soaked with 0.05% Tween-20 solution for 30–60 s, 5% sodium hypochlorite solution for 3–5 min, 2.5% sodium thiosulfate solution for 5–10 min, and 75% ethanol for 2–3 min, followed by multiple rinses with sterile water. In the end, the tissues were cut into small segments of approximately 1 cm, placed on the PDA medium supplemented with ampicillin sodium (0.1 mg/ml) and cultured at 28°C for 6–8 days. After the colonies grew in the incision of the tissues, they were transferred to fresh PDA medium for purification and preserved at 4°C for further use.

### Phylogenetic Analysis of Endophytic Fungi

The ITS rDNA sequences of the separated strains were amplified with universal ITS primers, ITS1 (5′-TCCGTAGGTGAACC TGCGG-3′) and ITS4 (5′-TCCTCCGCTTATTGATATGC-3′), and using an initial denaturation temperature of 94°C for 5 min, with 35 cycles of denaturation at 94°C for 30 s, annealing at 52°C for 30 s, extension at 72°C for 1 min, and a final extension of 72°C for 10 min.

The ITS sequencing was performed by Qingdao Ruibio Biotech Co., Ltd. All resulting ITS sequences were subjected to a BLAST search in the National Center for Biotechnology Information (NCBI) database to obtain closely related strains. A phylogenetic tree was generated with the neighbor-joining (NJ) method in the MEGA 7.0 software, and 1,000 replicates were used for the bootstrap analysis (Tamura et al., 2011).

### Fermentation and Bioactivity of Endophytic Fungi

All of the isolated strains were cultured in 400 ml ME medium for small-scale fermentation. The crude extracts were obtained by extracting the fermentation broth with ethyl acetate and dried by rotary evaporator. The crude extract was weighed and dissolved with methanol to prepare a solution of 20 mg/ml. Their antifungal and antibacterial activities were tested using the cup-plate method (Zaidan et al., 2005), which was based on the measurement of inhibition zones of microbial growth. First, a certain amount of sterile agar medium was poured into Petri dishes. After solidification, Oxford cups were placed equidistantly, and 15 ml LB medium mixed with pathogens (containing 1–5 × 10^7^ CFU/ml *S. aureus*, *S. enteritidis*, or spores of *F. graminearum*) was added. Subsequently, 50 μl of fermentation crude extract in methanol (20 mg/ml) was added to each well formed by Oxford cup. The plates inoculated with bacteria were cultured at 37°C for 24 h, and Petri dishes containing fungi were cultured at 28°C for 3 days. Then, the diameters of inhibition zones were measured. Ampicillin sodium (20 mg/ml) and actinomycin (20 mg/ml) were used as the positive control of antagonistic bacteria and fungi, respectively.

### Large-Scale Fermentation of Target Strain

The strain whose crude extract showed good activity and high yield was selected for large-scale fermentation. First, the strain was inoculated in 400 ml ME medium and shaken for 4 days at 28°C and 180 rpm. After that, the fermented broth was inoculated into 70 L ME medium in a 100 L fermenter for 10 days culture. The fermentation product was filtered with four layers of gauze to obtain fermentation supernatant and mycelium. The supernatant was added with equal volume of ethyl acetate and extracted three times. The mycelium was dried and extracted by methanol: dichloromethane (1:1) for three times. The solvents were evaporated *in vacuo* to give two dark-brown pastes, which were then merged into one crude extract for further chemical investigation.

### Activity Verification of Target Strain

After large-scale fermentation, a small amount of crude extract from the target strain was dissolved in methanol and used for antifungal activity verification. As detailed in previous literature (Xiang et al., 2018), the mycelial growth inhibition method was used with some modifications. The PDA media added with crude extract (500 μg/ml) was used as the experimental group, and the media mixed with 2% methanol was used as the control group. The pathogen disks (*F. graminearum*, *F. moniliforme*, *B. cinerea*, or *V. dahlia*) were placed in the middle of the test dishes and then placed in a constant temperature incubator at 28°C for 5–8 days. The colony diameters of pathogenic fungi were measured to calculate inhibition rates (Formula 1):


(1) $$\mathrm{Inhibition}\,\mathrm{rate}\%=\frac{A_{1}-A_{2}}{A_{1}-A_{0}}\,\times 100\%$$

where A_1_ represents the colony diameter of the control group (mm); A_2_ represents the colony diameter of the experimental group (mm); and A_0_ represents the diameter of the pathogen disk (mm).

### Purification and Characterization of Compounds

**FIGURE 1:**
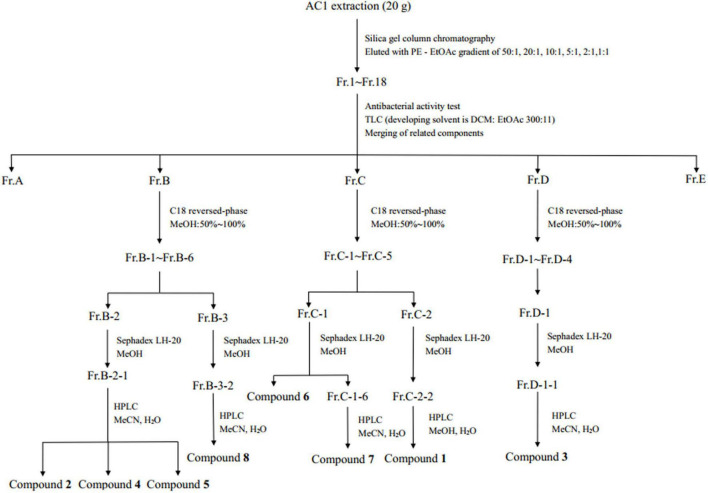
Work up schema of the endophytic fungus strain AC1.

The crude extract was eluted by silica gel column chromatography with gradient elution of PE-EtOAc, and the polarity of the eluent increased gradually (Figure 1). TLC was used to detect each fraction, and similar ones were combined to obtain five components (Fr.A–Fr.E). The components B, C, and D with antimicrobial activity were further separated by C18 reverse-phase silica gel column with the eluent of 50–100% methanol. The fractions were further separated by Sephadex LH-20 gel column and semi-preparative HPLC. Compounds **2** (6.1 mg), **4** (707 mg), **5** (194.19 mg), and **8** (10.9 mg) were received from Fr.B. Compounds **1** (19.7 mg), **6** (4.5 mg), and **7** (2.6 mg) were isolated from Fr.C. Compound **3** (139.9 mg) was isolated from Fr.D.

The structures of the compounds were identified using the nuclear magnetic resonance (NMR) spectrometer (Bruker Avance, Germany) and/or AB SCIEX X500R QTOF mass spectrometer. ^1^H NMR and ^13^C NMR spectra were measured in CDCl_3_ or acetone-*d*_6_ using tetramethylsilane (TMS) as the internal standard.

### Antimicrobial Activity of Compounds

According to the methods described in the literature (Abdalla et al., 2020), the inhibitory activities of monomeric compounds against four pathogenic fungi (*F. graminearum*, *F. moniliforme*, *B. cinerea*, and *V. dahliae*) and four bacteria (*S. aureus*, *E. coli*, *S. enteritidis*, and *P. aeruginosa*) were determined by microdilution method. First, 90 μl of the solution (containing 1–5 × 10^5^ CFU/ml bacteria or fungal spores) and 10 μl of the compounds (32–512 μg/ml) were added to each well. The bacteria and fungi were cultured at 37°C for 12 h and 28°C for 48 h, respectively. Subsequently, absorbance was measured at 600 nm, and the minimum inhibitory concentration (MIC) of the compound was determined with the absorbance value close to the blank value and no obvious turbidity in the pores as the standard. Ampicillin sodium and actinomycin were used as the positive control for antibacterial and antifungal tests, respectively. Each test was set up with three repeats.

### Cytotoxicity of Compounds

Hepatocellular carcinoma HepG2 cells, lung carcinoma A549 cells, and breast carcinoma MDA-MB-231 cells used in this study were purchased from Tongpai (Shanghai) Biotechnology Co., LTD. The cytotoxicities of compounds against the three kinds of human cancer cells were determined by MTT colorimetry assay using doxorubicin hydrochloride as the positive control (Huang et al., 2017). Cancer cells were added to a 96-well plate (100 μl/well) with a cell density of 4 × 10^4^ cell/ml and incubated at 37°C (5% CO_2_) for 20 h. Then, 100 μl of cell culture medium containing different concentrations of compounds (25–200 μM) was added into each well as the experimental group. The well added with 100 μl of cell culture medium without any compound was used as the negative control. After 48 h, the plate was washed once with PBST (150 μl/well) and added with MTT solution (1 mg/ml in DMEM containing 10% FBS, 100 μl/well) for another 4 h incubation. Then, the solution was removed, and DMSO (150 μl/well) was added to dissolve the methylzan crystals generated during incubation. After 10 min of shaking, the absorbance was measured at 570 nm. Each treatment was repeated three times. The inhibition rates of different concentrations of compounds were calculated by the following formula:


(2) $$\mathrm{Inhibition}\,\mathrm{rate}\%=\frac{A_{0}-A_{1}}{A_{0}}\,\times 100\%$$

where A_1_ represents the absorbance of the experimental group and A_0_ represents the absorbance of the negative control.

The 50% inhibitory concentration (IC_50_) was obtained using the Origine Pro 8.5 software.

## Results

### Isolation and Identification of Endophytic Fungi From *Artemisia argyi*

As shown in Table 1, a total of 26 endophytic fungi were isolated from *A. argyi* in this study. Two strains (AC1 and AC2) were isolated from roots, and 10 (AC3-AC12) and 16 (AC13-AC26) strains were separated from stems and leaves, respectively. The ITS rDNA sequences of all strains were sequenced and placed in NCBI with the accession numbers OL589639 and OM763739–OM763763. The ITS sequences were compared with available sequences already registered in the NCBI. According to the blast results, all sequences of these strains had no less than 99% similarity to the closest matches retrieved from the NCBI GenBank database, except for strain AC3, which showed a low identity of 96% with the sequence JN209922.1 (Table 1).

**TABLE 1:** The separation tissues and homologous strains of endophytic fungi from *A. argyi.*

Strains	Plant tissue	Closest sequences by BlastN	Coverage/max ident	Accession	Genbank
AC1	Root	*Diaporthe cotoneastri*	99/99.44	KJ609015.1	OL589639
AC2	Root	*Alternaria alternatim*	99/100.00	OK036714.1	OM763739
AC3	Stem	*Chaetomium* sp.	98/96.67	JN209922.1	OM763740
AC4	Stem	*Alternaria brassicae*	100/99.81	MF356574.1	OM763741
AC5	Stem	*Alternaria brassicae*	100/99.81	MN856410.1	OM763742
AC6	Stem	*Phoma* sp.	99/99.60	KX008381.1	OM763743
AC7	Stem	*Fusarium oxysporum*	98/100.00	MW599748.1	OM763744
AC8	Stem	*Alternaria alternata*	100/99.62	MK968044.1	OM763745
AC9	Stem	*Phoma* sp.	98/99.8	MW784633.1	OM763746
AC10	Stem	*Alternaria* sp.	100/100.00	MK649975.1	OM763747
AC11	Stem	*Trichoderma asperellum*	100/100.00	MK841019.1	OM763748
AC12	Stem	*Gibberella avenacea*	97/99.62	EU255800.1	OM763749
AC13	Leaf	*Fusarium petersiae*	97/99.43	MG386079.1	OM763750
AC14	Leaf	*Chaetomium globosum*	96/99.44	GU138648.1	OM763751
AC15	Leaf	*Fusarium falciforme*	99/99.81	MT251175.1	OM763752
AC16	Leaf	*Alternaria tenuissima*	100/99.44	KF919124.1	OM763753
AC17	Leaf	*Alternaria tamaricis*	100/99.81	OM236747.1	OM763754
AC18	Leaf	*Fusarium tricinctum*	97/99.62	MT446111.1	OM763755
AC19	Leaf	*Fusarium* sp.	94/99.22	MK645987.1	OM763756
AC20	Leaf	*Colletotrichum gloeosporioides*	98/100	JN887348.1	OM763757
AC21	Leaf	*Diaporthe phaseolorum*	99/99.26	KP743032.1	OM763758
AC22	Leaf	*Alternaria porri*	99/100	MN872486.1	OM763759
AC23	Leaf	*Alternaria solani*	100/99.81	OM236915.1	OM763760
AC24	Leaf	*Fusarium verticillioides*	99/99.41	MH729015.1	OM763761
AC25	Leaf	*Fusarium avenaceum*	98/99.81	KY272764.1	OM763762
AC26	Leaf	*Fusarium arcuatisporum*	99/99.60	MW016518.1	OM763763

**FIGURE 2:**
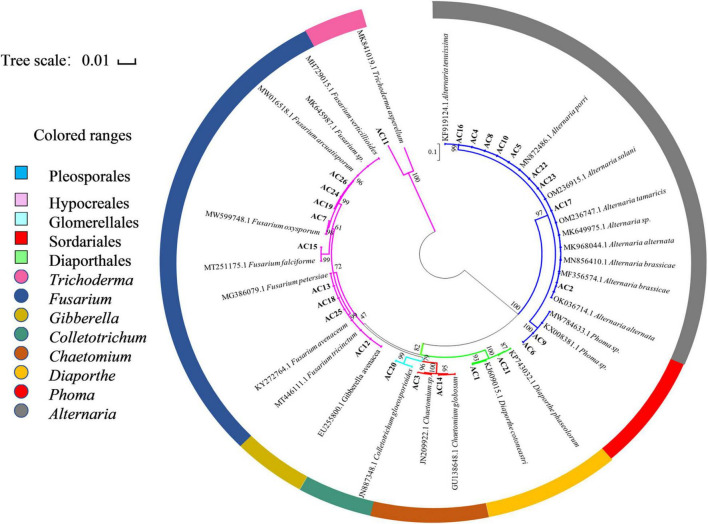
Neighbor-joining phylogenetic tree based on ITS sequences of 26 endophytic strains. Numbers at the branch points are the bootstrap values based on 1,000 resamplings. The scale bar represents 0.01 nucleotide changes per position.

Based on the analysis of homology of the ITS rDNA sequences, a phylogenetic relationship was constructed by the neighbor-joining method (Figure 2). All the isolated fungi belonged to one phylum (Ascomycota), two classes (Dothideomycetes and Sordariomycetes), five orders (Pleosporales, Hypocreales, Glomerellales, Sordariales, and Diaporthales), and eight genera [*Alternaria* (34.62%), *Fusarium* (30.77%), *Chaetomium* (7.69%), *Phoma* (7.69%), *Diaporthe* (7.69%), *Trichoderma* (3.85%), *Gibberella* (3.85%), and *Colletotrichum* (3.85%)], demonstrating the rich biodiversity of endophytic fungi from *A. argyi*.

### Yields and Antimicrobial Activities of the Crude Extracts

The yields and antimicrobial activities of crude extracts from *A. argyi*-associated endophytes are shown in Table 2. The crude extract yield of strain AC7 was the highest (1,180 mg/L), followed by AC14 (580 mg/L) and AC1 (474 mg/L).

**TABLE 2:** Yields and antimicrobial activities of the crude extracts from endophytic fungi.

Strain	Total weight of extract (mg/L)	Inhibitory zone diameter/mm		
		
		*S. aureus*	*S. enteritidis*	*F. graminearum*
AC1	474	13.8	21.8	12.5
AC2	188	11.5	19.7	−
AC3	211	−	13.3	A
AC4	311	12.3	13.3	14.5
AC5	371	13.0	19.8	19.0
AC6	143	12.3	20.3	12.5
AC7	1,180	13.3	19.3	−
AC8	171	14.0	18.7	11.8
AC9	57	−	12.3	−
AC10	354	12.8	22.3	19.0
AC11	298	13.3	14.3	16.0
AC12	357	−	−	a
AC13	379	14.0	15.3	a
AC14	580	−	20.5	15.5
AC15	31	12.8	−	a
AC16	314	15.3	21.3	13.3
AC17	186	13.8	19.5	a
AC18	201	13.7	14.8	a
AC19	209	12.3	15.8	−
AC20	187	12.8	−	−
AC21	169	13.0	−	−
AC22	57	12.5	18.5	−
AC23	176	−	−	a
AC24	378	−	−	16.0
AC25	236	−	−	18.0
AC26	361	−	−	−
Ampicillin sodium		36.7	40.0	n
Actinomycin		n	n	27.5

*“−” means no inhibition. “a” means there was inhibitory effect, but the boundary of the inhibition zone was not clear. “n” means not tested.*

All isolates except AC26 had inhibitory effects on at least one of the three tested pathogens. Among them, 11 strains (i.e., AC1, AC4-AC6, AC8, AC10, AC11, AC13, and AC16-AC18) showed inhibitory activity against all three tested strains, and 7 strains (i.e., AC2, AC3, AC7, AC14, AC15, AC19, and AC22) could inhibit two tested pathogens. The endophytic fungi AC1, AC6, AC10, AC14, and AC16 exhibited an obvious inhibitory effect on pathogenic bacteria *S. enteritidis* with the diameter of inhibition zone more than 20 mm. Strains AC5 and AC10 showed a good inhibition effect on pathogenic fungus *F. graminearum*, and the inhibition zone diameter was 19 mm. The inhibition zones generated by crude extracts of endophytic fungi can be seen in Supplementary Figure 3. The crude extracts inhibition zones against bacteria had clear boundaries, but some of that against pathogenic fungi did not show clear boundaries, including that generated by the crude extracts of AC3, AC12, AC13, AC15, AC17, AC18, and AC23. It could be explained that these crude extracts could not completely inhibit the growth of pathogenic fungi or had weak inhibition to pathogenic fungi.

Combined with the yields and activities of crude extracts, strain AC1 was selected for large-scale fermentation. Finally, AC1 was characterized as *Diaporthe* sp. at the genus level based on the observation of colony morphology (Supplementary Figure 4) and phylogenetic analysis (Supplementary Figure 5).

### Activity Verification of Crude Extract From *Diaporthe* sp. AC1

**FIGURE 3:**
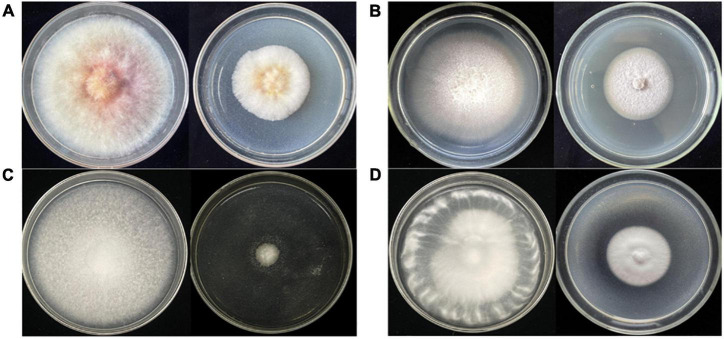
Antifungal activity of the crude extract from strain AC1. **(A)** *F. graminearum*; **(B)** *F. moniliform*e; **(C)** *B. cinerea*; **(D)** *V. dahlia* (left: control group; right: experimental group).

After large-scale fermentation, a total of 20 g crude extract was obtained. The crude extract was subjected to an antifungal assay to verify its activity. As shown in Figure 3, the crude extract of *Diaporthe* sp. AC1 displayed inhibitory activity against the four pathogenic fungi, namely, *F. graminearum*, *F. moniliform*e, *B. cinerea*, and *V. dahlia* with inhibition rates of 44.2, 38.7, 86.8, and 49.9%, respectively. Then, the crude extract of the strain AC1 was isolated and purified through multiple chromatographic techniques.

### Elucidation of Compounds From *Diaporthe* sp. AC1

**FIGURE 4:**
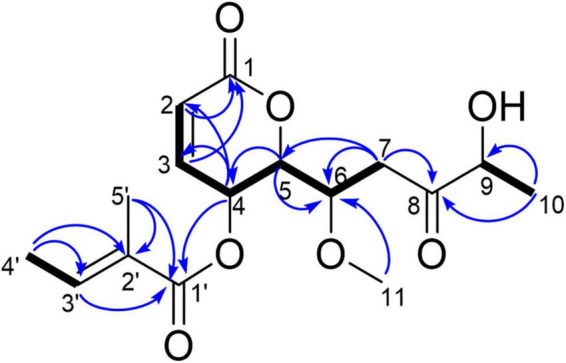
Key HMBC (blue arrow) and ^1^H–^1^H COSY correlations (black bond) of **1**.

Compound **1**, light yellow powder. ^1^H NMR (400 MHz, CDCl_3_) and ^13^C NMR (101 MHz, CDCl_3_) (see Table 3); positive HR-MS m/z 349.1258 [M + Na]^+^ (calculated for C_16_H_22_O_7_Na, 349.1263). HR-MS of compound **1** indicated a molecular formula of C_16_H_22_O_7_. Comparison of the NMR data of **1** with those of compound **2** (phomopsolide F) revealed that **1** had a methoxy group at δ_*C*_ 59.04 (Table 3). This data indicated that one of the two hydroxyl groups was methylated. Further analysis of correlations in the ^1^H–^1^H COSY and HMBC spectra demonstrated that the hydroxyl group at C_6_ was methylated (Figure 4). Thus, compound **1** was determined as a methyl derivative of phomopsolide F and named as phomopsolide G (NMR and HR-MS data can be found in Supplementary Figures 6–12).

**TABLE 3:** ^13^C-NMR and ^1^H-NMR data of compounds **1** and **2**.

	δ_*C*_		δ_*H*_ mult (*J*)	
		
No.	1	2	1	2
1	161.92	162.00		
2	124.74	124.75	6.22 (d, 9.6 Hz, 1 H)	6.25 (d, 9.7 Hz, 1 H)
3	141.26	140.52	7.16 (dd, 9.6, 5.9 Hz, 1 H)	7.04 (dd, 9.7, 5.8 Hz, 1 H)
4	60.87	67.03	5.42 (dd, 6.0, 2.5 Hz, 1 H)	5.45 (dd, 5.9, 2.7 Hz, 1 H)
5	79.27	80.43	4.45 (dd, 9.1, 2.5 Hz, 1 H)	4.58 (dd, 6.8, 2.7 Hz, 1 H)
6	74.32	61.59	4.18 (m, 1 H)	4.26 (q, 7.1 Hz, 1 H)
7	39.32	39.29	3.05 (dd, 16.9, 4.1 Hz, 1 H) 2.88 (dd, 16.9, 6.6 Hz, 1 H)	2.81 (dd, 16.1, 3.8 Hz, 1 H) 2.75 (dd, 16.2, 7.4 Hz, 1 H)
8	210.41	211.00		
9	73.16	73.68	4.37 (q, 7.2 Hz, 1 H)	4.56–4.48 (m, 1 H)
10	19.35	19.18	1.43 (d, 7.12 Hz, 3 H)	1.38 (d,7.1 Hz, 3 H)
**11**	**59.04**		**3.32** (**s, 1 H**)	
1′	166.47	166.57		
2′	127.54	127.36		
3′	139.96	140.43	6.98–6.92 (m, 1 H)	6.96–6.81 (m, 1 H)
4′	12.09	12.06	1.86–1.79 (m, 6 H)	1.86–1.79 (m, 6 H)
5′	14.67	14.69		

*Bold values can make it easier for the reader to see the difference between the two compounds.*

**FIGURE 5:**
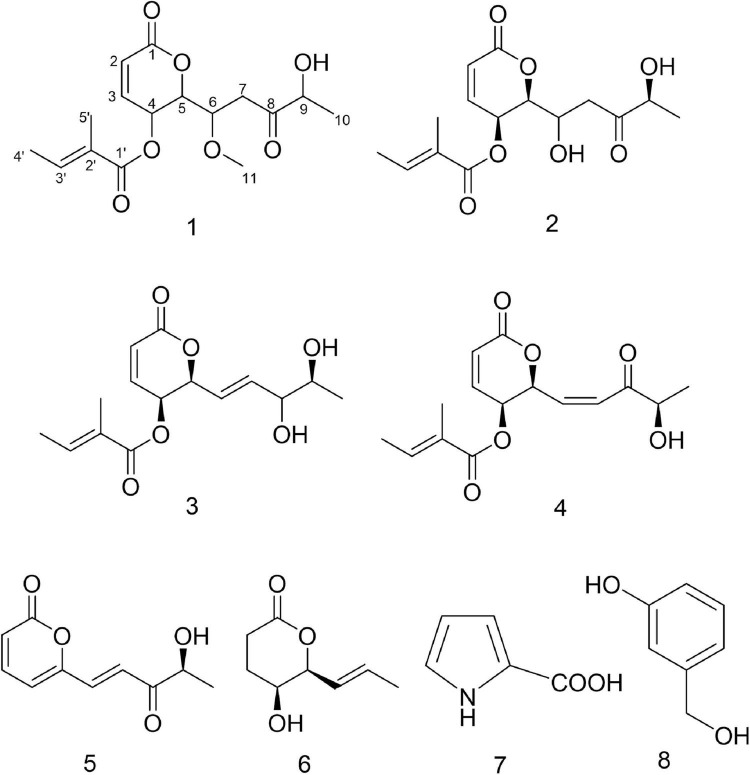
Structures of compounds **1–8**.

Compound **2**, reddish-brown oil. ^1^H NMR (400 MHz, CDCl_3_) and ^13^C NMR (101 MHz, CDCl_3_) (see Table 3 and Supplementary Figures 13, 14). These data indicated that the compound was the same as phomopsolide F (Stierle et al., 2014; Figure 5).

The NMR data of compounds **3**–**8** in Supplementary Figures 15–26 allowed the identification of phomopsolide B (**3**) and A (**4**) (Stierle et al., 1997, 2014), (S,E)-6-(4-hydroxy-3-oxopent-1-en-1-yl)-2H-pyran-2-one (**5**) (Grove, 1985; Tanney et al., 2016), catenioblin A (**6**) (Wu et al., 2012), 2-minaline (**7**) (Wang et al., 2014), and 3-hydroxybenzyl alcohol (**8**) (Geng et al., 2012). The structures of compounds **3**–**8** are shown in Figure 5.

### Antimicrobial Activity of Compounds From *Diaporthe* sp. AC1

The antimicrobial activities of compounds **1–8** against pathogenic fungi and bacteria were evaluated by determining their MIC values. According to Table 4, compounds **4**, **5,** and **7** could inhibit all of the four pathogenic fungi, and the new compound **1** had antagonistic effect on three fungi (*F. graminearum*, *F. moniliforme*, and *B. cinerea*) with MIC values smaller than or equal to 512 μg/ml. Especially, the MIC values of compound **5** against *F. graminearum* and *B. cinerea* were both 128 μg/ml, which were close to the positive control (64 and 128 μg/ml, respectively).

**TABLE 4:** Antifungal activity of compounds **1–8** and cycloheximide.

Compounds	Pathogenic fungi (MIC μg/ml)			
	
	*F. graminearum*	*F. moniliforme*	*B. cinerea*	*V. dahliae*
**1**	256	512	256	−
**2**	−	−	−	−
**3**	512	−	−	−
**4**	256	256	512	512
**5**	128	256	128	512
**6**	−	−	−	−
**7**	512	256	512	256
**8**	−	−	−	−
Actinomycin	64	128	128	128

*“−” means no inhibition.*

As shown in Table 5, compounds **4** and **5** have weak inhibitory effect on four kinds of pathogenic bacteria with MIC values less than or equal to 512 μg/ml. It can also be seen that compound **7** could inhibit *S. aureus* and *P. aeruginosa*, compound **3** could inhibit *S. aureus* and *P. aeruginosa*, while compounds **1**, **2,** and **6** could only inhibit *S. aureus* with MIC values less than or equal to 512 μg/ml.

**TABLE 5:** Antibacterial activity of compounds **1–8** and ampicillin sodium.

Compounds	Pathogenic bacteria (MIC μg/ml)			
	
	*S. aureus*	*E. coli*	*S. enteritidis*	*P. aeruginosa*
**1**	256	−	b	−
**2**	512	−	b	−
**3**	64	b	b	512
**4**	64	512	512	256
**5**	256	256	512	256
**6**	512	−	−	−
**7**	256	−	−	256
**8**	−	−	−	−
Ampicillin sodium	2	64	32	128

*“−” means no inhibition. “b” means there was antibacterial effect but MIC > 512 μg/ml.*

### Cytotoxicity Assay of Compounds From *Diaporthe* sp. AC1

**FIGURE 6:**
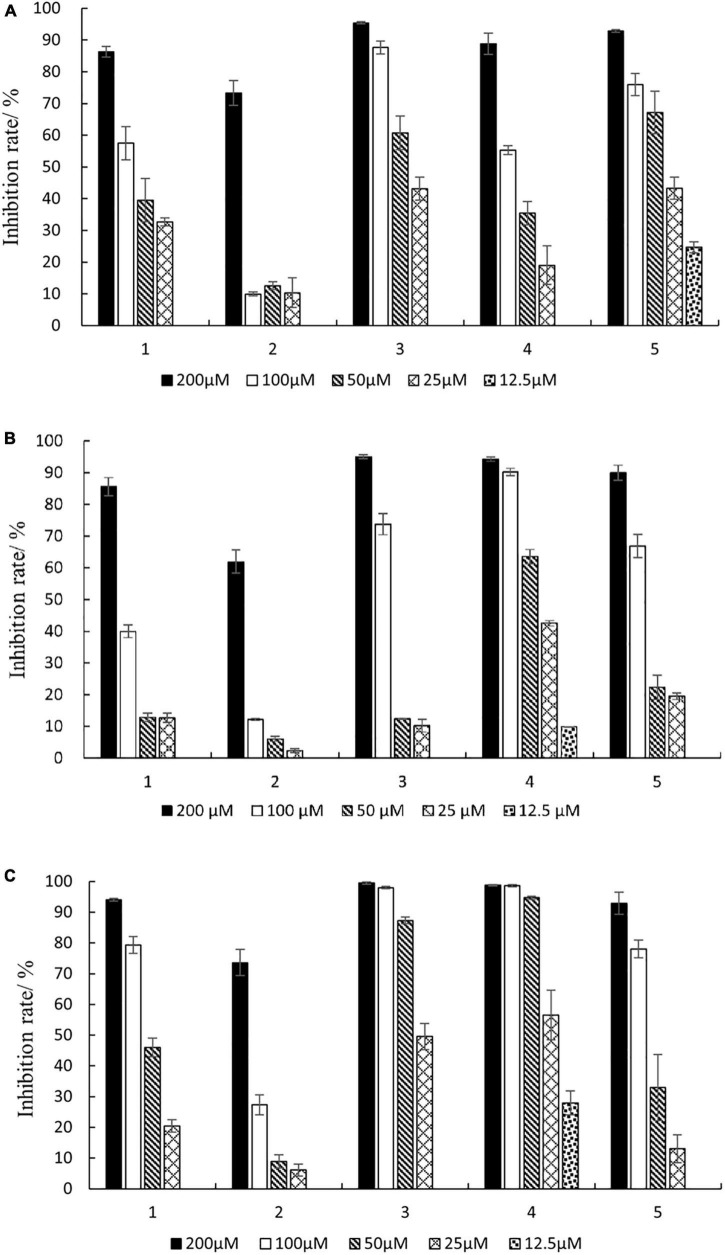
Inhibition rates against three cancer cells of different concentrations of compounds **1–5**. **(A)** HepG2; **(B)** A549; **(C)** MDA-MB-231.

As shown in Figure 6, compounds **1–5** had inhibitory activity on three kinds of carcinoma cells, and the inhibition rate increased with the increase of compound concentration. The inhibition rates of compounds **1–5** ranged from 63.8 to 99.5% at a concentration of 200 μM. Furthermore, the IC_50_ values of the compounds were calculated (Supplementary Table 1). The results indicated that the IC_50_ values ranged from 23.06 to 183.92 μM. The new compound **1** (phomopsolide G) displayed weak cytotoxicity against HepG2, A549, and MDA-MB-231, with IC_50_ values of 89.91, 107.65, and 53.97 μM, respectively. Compound **3** showed moderate cytotoxicity against HepG2 and MDA-MB-231 with IC_50_ values of 36.71 and 26.21 μM, respectively. Compound **4** exhibited moderate cytotoxicity to A549 and MDA-MB-231 with IC_50_ values of 36.54 and 23.06 μM, respectively. In addition, compound **5** could inhibit the growth of HepG2 with IC_50_ value of 30.11 μM.

## Discussion

Endophytes were increasingly reported to be important sources of bioactive compounds in the past decade (Charria-Girón et al., 2021). Additionally, several studies involving endophytic fungi derived from medicinal plants have led to the discovery of novel compounds with attractive pharmaceutical applications (Kaul et al., 2012; Adeleke and Babalola, 2021). *A. argyi* is a traditional Chinese herbal medicine plant, and a study on the diversity of its endophytic community has been reported (Wu et al., 2021). In that study, the endophytic fungal communities of *A. argyi* from different regions were analyzed by amplicon sequencing and culture-free technology. Although culture-free technology can provide a lot of species information, it cannot reveal the bioactive functions of endophytes. Traditional culture-dependent technology can make us fully understand the characteristics of microorganisms and lay a foundation for the development of biological agents. In this study, the cultivable endophytic fungi associated with *A.* argyi were studied. Twenty-six fungi, which belonged to one phylum and two classes, were isolated by culture-dependent method and molecular biological identification. What was worth mentioning was that AC3 strain showed a sequence match of 96% with *Chaetomium* sp. (JN209922.1), which indicated that it might be a potential new species (Landeweert et al., 2003).

Nowadays, antibiotic resistance has been one of the greatest challenges in the health system and has urged the need for new compounds with broad-spectrum bioactivity to treat infectious diseases (Vivas et al., 2019). *S. aureus* is a major pathogen that causes a wide variety of community and hospital-acquired infectious diseases (Tong et al., 2015). The frequency of these infections is increasing, and the treatment is becoming more difficult due to the emergence of multiple drug-resistant (MDR) strains (Oliveira et al., 2018). Foodborne infectious disease is also a major public health concern worldwide with countries expending many resources to overcome it. *Salmonella* is one of the most frequently isolated foodborne pathogens, accounting for more than half of the numbers of foodborne outbreak illnesses reported in the EU (Ehuwa et al., 2021). Moreover, the emergence of MDR *Salmonella* strains poses a great challenge in terms of effective treatment of the infections caused by these strains and may lead to an increase in mortality rates of *Salmonella* infections (Eng et al., 2015). Therefore, the Gram-positive *S. aureus* and Gram-negative *S. enteritidis* were selected as the test strain used in preliminary screening.

Fusarium head blight (FHB), mainly caused by *F. graminearum*, is one of the most important cereal diseases worldwide due to its significant reductions in grain yield and quality (Torres et al., 2019). Over the last four decades, chemical fungicides have been used to control the risk of FHB and can effectively reduce disease incidence. However, repeated applications of fungicides can lead to fungicide resistance (Wegulo et al., 2015). In recent years, many endophytic fungi have displayed biocontrol potential against FHB (Comby et al., 2017; Rojas et al., 2020; Abaya et al., 2021). Hence, the antifungal activity against *F. graminearum* of endophytic fungi from *A. argyi* was tested in this study.

The screening of antimicrobial activity using crude extracts allows the identification of potential producer strains and the further isolation of compounds with biological activity. In this context, crude extracts of the 26 endophytic fungi were screened for antimicrobial activity. Among them, 25 crude extracts had antimicrobial activity, exhibiting a high proportion of biological activity. Moreover, since bioactive molecules are often present in secondary metabolites in minimal quantities, high yields of crude extracts are necessary for the study of bioactive natural products. Among the 26 endophytic fungi, *Diaporthe* sp. AC1 had high yield and good inhibitory effect against the tested pathogens and thus was fermented for further study. *Diaporthe* spp. are distributed widely in many plants and have been regarded as potential sources for producing diverse bioactive metabolites (Xu et al., 2021). Most of the compounds derived from this genus are polyketides, exhibiting various bioactivities, including cytotoxic (Carvalho et al., 2018), antifungal (Luo et al., 2018), antibacterial (Niaz et al., 2021), antiviral (Yang et al., 2020), antioxidant (Da Rosa et al., 2020), and anti-inflammatory activities (Fan et al., 2020).

Chemical investigation of the crude extract of *Diaporthe* sp. AC1 led to the discovery of phomopsolides A (**4**), B (**3**), F (**2**), and G (**1**), among which phomopsolide G was a new compound. Phomopsolides are common secondary metabolites derived from *Diaporthe* (Xu et al., 2021) and have been proved to have antimicrobial activity. Especially, phomopsolide A (**4**) was reported to show good antifungal activity against *Microbotryum violaceum*, nice antibacterial activity against *Saccharomyces cerevisiae* and *Bacillus subtilis*, and moderate inhibitory activity against *Mycobacterium tuberculosis* (Stierle et al., 1997; Clark et al., 2015; Tanney et al., 2016). In this study, phomopsolide A was proved to inhibit the growth of *F. graminearum*, *F. moniliforme*, *B. cinerea*, and *V. dahliae*, indicating that the compound may have a wide spectrum of antifungal activity. The new compound phomopsolide G (**1**) showed moderate antifungal activity against *F. graminearum*, *F. moniliforme*, and *B. cinerea* and weak antibacterial activity against *S. aureus.* The other four compounds were (S, E)-6-(4-hydroxy-3-oxopent-1-en-1-yl)-2H- pyran-2-one (**5**), catenioblin A (**6**), 2-minaline (**7**), and 3-hydroxybenzyl alcohol (**8**). Compound **5** showed moderate antifungal activity against the four tested plant pathogenic fungi and weak antibacterial activity against the four bacteria. To our best knowledge, the antimicrobial activity of compound **5** was first reported in this study. Compound **7** also exhibited moderate antifungal activity against the four tested plant pathogenic fungi and weak antibacterial activity against *S. aureus* and *P. aeruginosa*.

Cancer is currently a major cause of death in most countries of the world (Bray et al., 2021; Ferlay et al., 2021). Plant endophytic fungi have been recognized as an important resource of anticancer compounds (Kumar et al., 2021; Hridoy et al., 2022). Many of these anticancer compounds, such as paclitaxel, vinblastine, camptothecin, and podophyllotoxin, are currently being used clinically for the treatment of various cancers. In this study, the cytotoxicity of the compounds isolated from *Diaporthe* sp. AC1 was measured. Phomopsolide A (**4**) exhibited cytotoxicity against HepG2, A549, and MDA-MB-231 with IC_50_ values of 84.0, 36.7, and 23.0 μM, respectively. In previous study, phomopsolide A has been proved to have good inhibitory activity against cervical adenocarcinoma HeLa S3, bladder transitional cell carcinoma UMUC-3, and embryonic kidney 293 cells with IC_50_ values under 10 μM (Stierle et al., 2014; Clark et al., 2015). These collectively demonstrated that phomopsolide A might be a broad-spectrum antitumor compound. In addition, the new compound phomopsolide G (**1**) displayed weak cytotoxicity against HepG2, A549, and MDA-MB-231, with IC_50_ values of 79.8, 107.5, and 54.0 μM, respectively.

## Conclusion

In conclusion, 26 endophytic fungi were isolated and identified from *A. argyi*, which belonged to eight genera. The antimicrobial assay demonstrated that 25 fungal extracts (96%) exhibited inhibitory activities against at least one of the tested pathogenic strains. Furthermore, an endophytic fungus *Diaporthe* sp. AC1 with good antimicrobial activity was selected for large-scale fermentation. Chemical investigation of this strain obtained eight compounds, including a new compound phomopsolide G (**1**) and seven known ones, namely, phomopsolide F (**2**), phomopsolide B (**3**), phomopsolide A (**4**), (S, E)-6-(4-hydroxy-3-oxopent-1-en-1-yl)-2H-pyran-2-one (**5**), catenioblin A (**6**), 2-minaline (**7**), and 3-hydroxybenzyl alcohol (**8**). The new compound **1** had weak antifungal, antibacterial, and cytotoxic activities, and other compounds also showed one or more activities. These results collectively demonstrated that endophytic fungi of *A. argyi* is an abundant source of new bioactive products with medicinal use and agricultural value.

## Data Availability Statement

The datasets presented in this study can be found in online repositories. The names of the repository/repositories and accession number(s) can be found below: https://www.ncbi.nlm.nih.gov/genbank/, OL589639, OM763739–OM763763.

## Author Contributions

HG, SZ, and LL performed the experiments and analyzed the data. ZY, FZ, and YT designed the experiments and edited the manuscript. All authors commented on previous versions of the manuscript and read and approved the final manuscript.

## Conflict of Interest

The authors declare that the research was conducted in the absence of any commercial or financial relationships that could be construed as a potential conflict of interest.

## Publisher’s Note

All claims expressed in this article are solely those of the authors and do not necessarily represent those of their affiliated organizations, or those of the publisher, the editors and the reviewers. Any product that may be evaluated in this article, or claim that may be made by its manufacturer, is not guaranteed or endorsed by the publisher.
