# Supplementary material for: Antimicrobial Potential of Endophytic Fungi From Artemisia argyi and Bioactive Metabolites From Diaporthe sp. AC1
Source: Front Microbiol. 2022 Jun 23;13:908836. doi: 10.3389/fmicb.2022.908836 (PMC9260665; doi:10.3389/fmicb.2022.908836)
Supplement: Supplementary file 1 [file Data_Sheet_1.doc]

Supplementary Material:

**Antimicrobial potential of endophytic fungi from *Artemisia argyi* and bioactive metabolites from *Diaporthe* sp. AC1**

**Haiping Gu^1^, Shikai Zhang^1^, Lin Liu^1^, Zhengyou Yang^1^,**^*^**, Fengchun Zhao^1,*^, Yuan Tian^2,*^**

1. Department of Microbiology, College of Life Science, Key Laboratory for Agriculture Microbiology, Shandong Agricultural University, Taian 271018, China
2. College of Life Science, Shandong First Medical University & Shandong Academy of Medical Sciences, Taian 271016, China

^*^Corresponding Author
Zhengyou Yang [zhyouyang@sdau.edu.cn](mailto:zhyouyang@sdau.edu.cn)

Fengchun Zhao [zhaofengchun@sdau.edu.cn](mailto:zhaofengchun@126.com)

Yuan Tian [tianyuan2005hit@163.com](mailto:tianyuan2005hit@163.com)

**Figure S1.** Microscopic morphology of four kinds of pathogenic fungi.

**Figure S2.** Neighbor-joining phylogenetic tree based on ITS sequences of four pathogenic fungi and relative strains.

**Figure S3.** Antimicrobial effect of crude extracts of endophytic fungi from *A. argyi.* (**A**): *S. aureus*; (**B**): *S. enteritidis*; (**C**): *F. graminearum*.

**Figure S4.** Colony morphology of AC1 strain after 5 (A) and 10 (B) days of culture.

**Figure S5.** Neighbor-joining phylogenetic tree based on ITS sequences of AC1 and relative strains.

**Figure S6.** The ^1^H NMR spectrum of phomopsolide G (**1**) (CDCl_3_, 400 MHz).

**Figure S7.** The ^13^C NMR spectrum of phomopsolide G (**1**) (CDCl_3_, 101 MHz).

**Figure S8.** The ^1^H–^1^H COSY spectrum of phomopsolide G (**1**) (CDCl_3_, 400 MHz).

**Figure S9.** The HSQC spectrum of phomopsolide G (**1**) (CDCl_3_, 400 MHz).

**Figure S10.** The HMBC spectrum of phomopsolide G (**1**) (CDCl_3_, 400 MHz).

**Figure S11.** The NOESY spectrum of phomopsolide G (**1**) (CDCl_3_, 400 MHz).

**Figure S12.** The HR-MS of phomopsolide G (**1**).

**Figure S13.** The ^1^H NMR spectrum of phomopsolide F (**2**) (CDCl_3_, 400 MHz).

**Figure S14.** The ^13^C NMR spectrum of phomopsolide F (**2**) (CDCl_3_, 101 MHz).

**Figure S15.** The ^1^H NMR spectrum of phomopsolide B (**3**) (CDCl_3_, 400 MHz).

**Figure S16.** The ^13^C NMR spectrum of phomopsolide B (**3**) (CDCl_3_, 101 MHz).

**Figure S17.** The ^1^H NMR spectrum of phomopsolide A (**4**) (CDCl_3_, 400 MHz).

**Figure S18.** The ^13^C NMR spectrum of phomopsolide A (**4**) (CDCl_3_, 101 MHz).

**Figure S19.** The ^1^H NMR spectrum of (S,E)-6-(4-hydroxy-3-oxopent-1-en-1-yl)-2H-pyran-2-one (**5**) (CDCl_3_, 400 MHz).

**Figure S20.** The ^13^C NMR spectrum of (S,E)-6-(4-hydroxy-3-oxopent-1-en-1-yl)-2H-pyran-2-one (**5**) (CDCl_3_, 101 MHz).

**Figure S21.** The ^1^H NMR spectrum of catenioblin A (**6**) (Acetone-*d*_6_, 400 MHz).

**Figure S22.** The ^13^C NMR spectrum of catenioblin A (**6**) (Acetone-*d*_6_, 101 MHz).

**Figure S23.** The ^1^H NMR spectrum of 2-minaline (**7**) (Acetone-*d*_6_, 400 MHz).

**Figure S24.** The ^13^C NMR spectrum of 2-minaline A (**7**) (Acetone-*d*_6_, 101 MHz).

**Figure S25.** The ^1^H NMR spectrum of 2-minaline (**8**) (Acetone-*d*_6_, 400 MHz).

**Figure S26.** The ^13^C NMR spectrum of 2-minaline A (**8**) (Acetone-*d*_6_, 101 MHz).

**TABLE S1** Cytotoxicity of compounds **1~5** and doxorubicin.


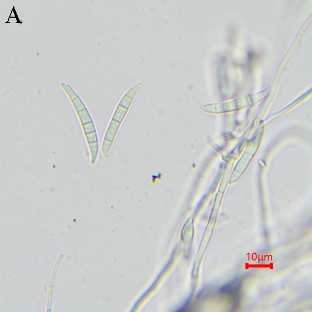

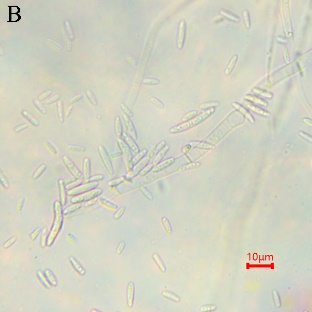

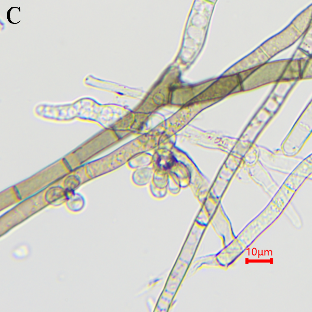

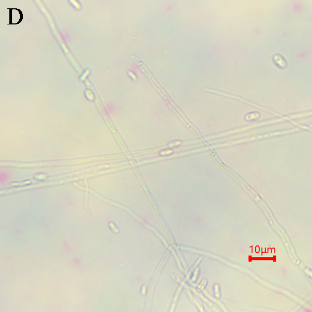


**Figure S1.** Microscopic morphology of four kinds of pathogenic fungi. (**A**): *F. graminearum*; (**B**): *F. moniliform*e; (**C**): *B. cinerea*; (**D**): *V. dahlia.*


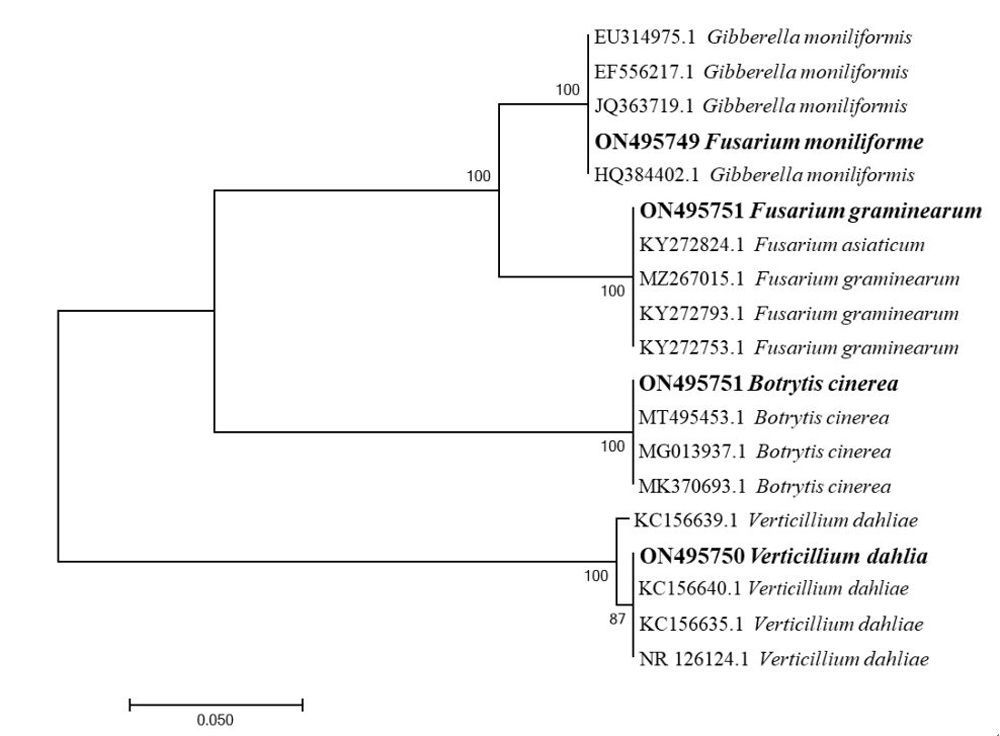


**Figure S2.** Neighbor-joining phylogenetic tree based on ITS sequences of four pathogenic fungi and relative strains. The pathogenic fungi used in this study are in bold. *Gibberella moniliformis* is the teleomorph of *Fusarium moniliformis*.


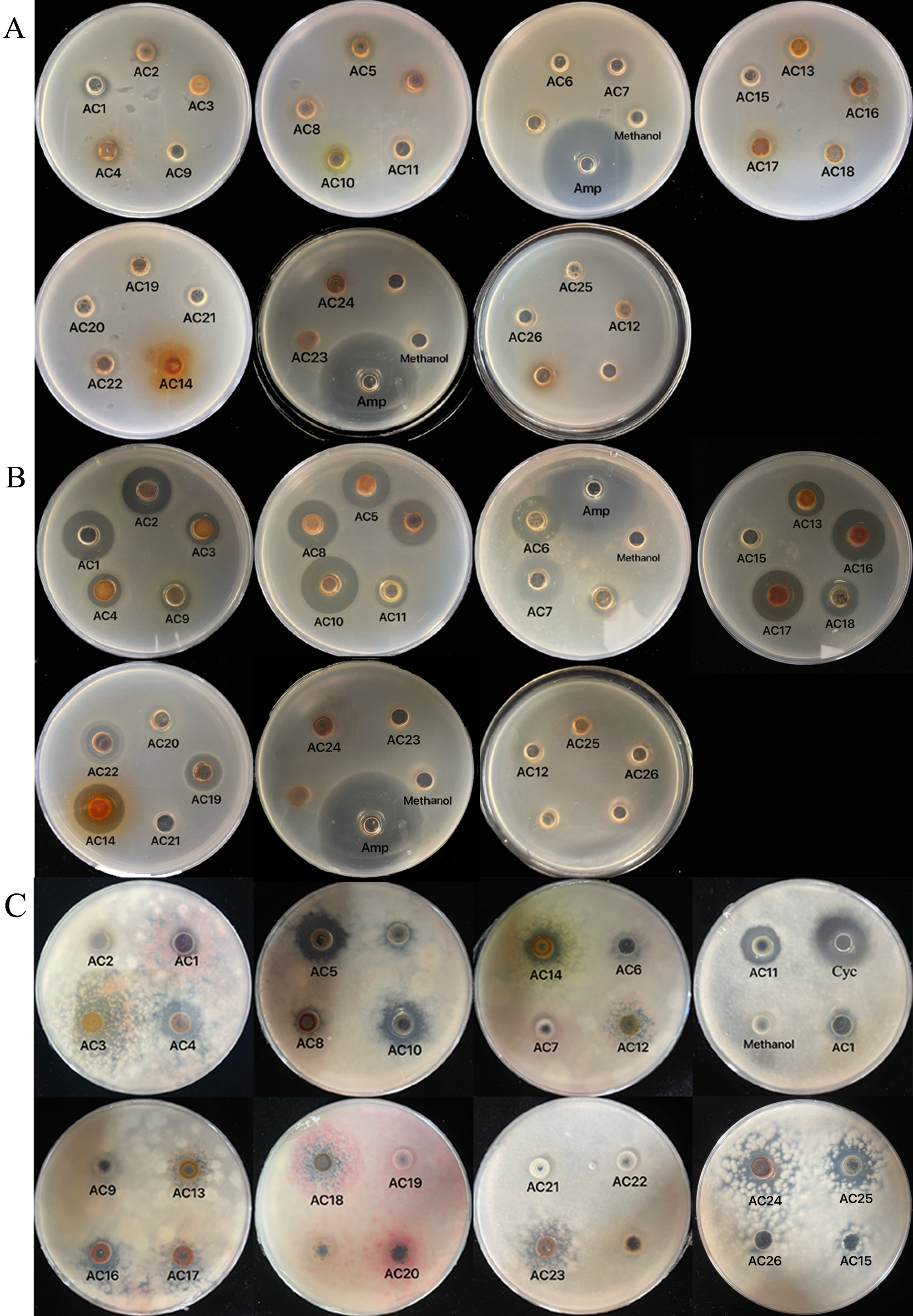


**Figure S3.** Antimicrobial effect of crude extracts of endophytic fungi from *A. argyi.* (**A**): *S. aureus*; (**B**): *S. enteritidis*; (**C**): *F. graminearum*.

(**A**)
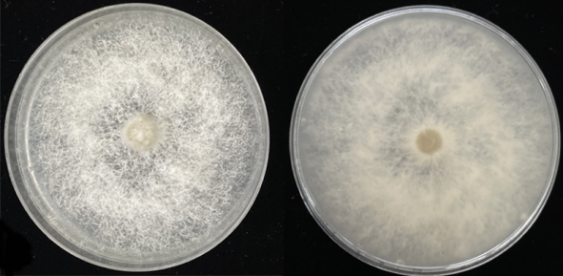
 (**B**)
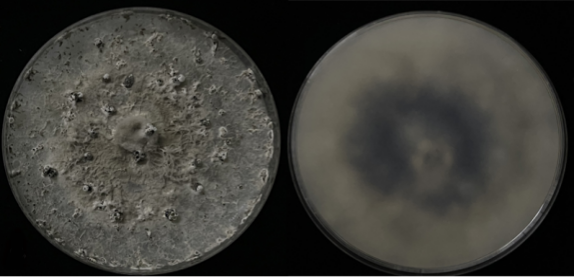


**Figure S4.** Colony morphology of AC1 strain after 5 (A) and 10 (B) days of culture.

*Diaporthe celastrina* CBS 139.27^T^ (NR 152457.1)

*Diaporthe alleghaniensis* CBS 495.72^T^ (NR 103696.1)

*Diaporthe cotoneastri* CBS 439.82^T^ (NR 119726.1)

*Diaporthe celeris*^T^ (NR 158433.1)

*Diaporthe rosicola* MFLU 17-0646^T^ (NR 157515.1)

AC1

*Diaporthe phragmitis*^T^ (NR 137927.1)

90

84

62

60

0.0020

**Figure S5.** Neighbor-joining phylogenetic tree based on ITS sequences of AC1 and relative strains.

Numbers at the branch points are the bootstrap values based on 1000 resamplings. Bootstrap values of above 35% are shown at branch points. The scale bar represents 0.0020 nucleotide changes per position.


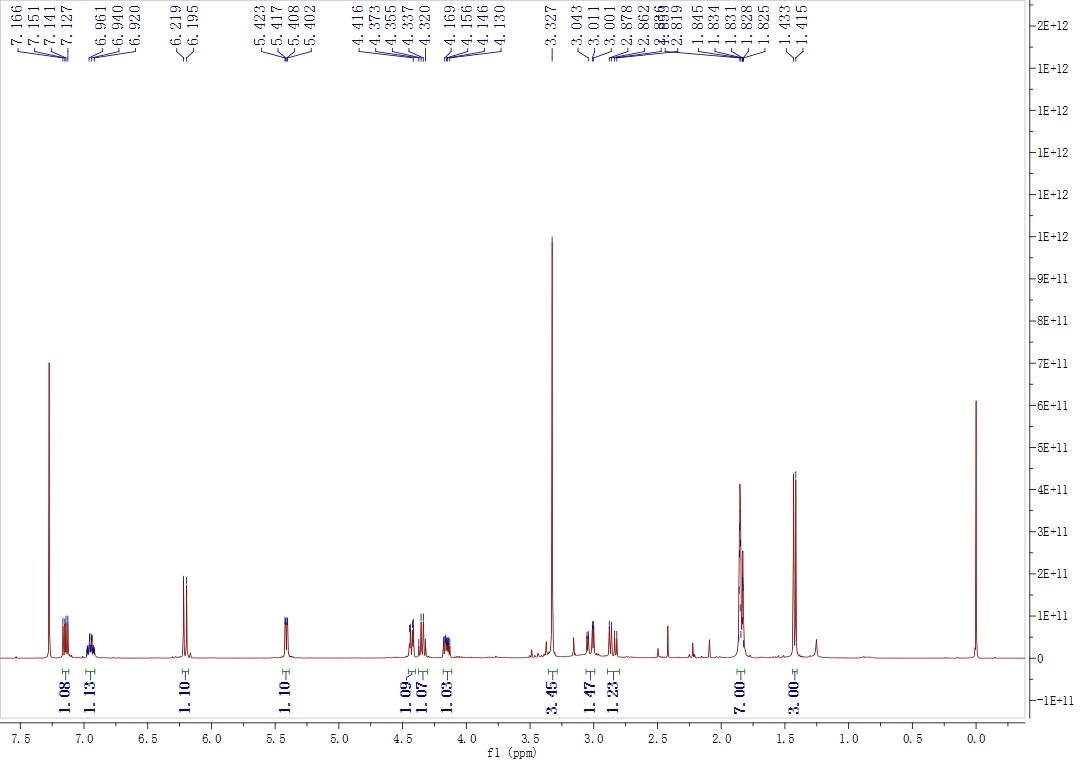


**Figure S6.** The ^1^H NMR spectrum of phomopsolide G (**1**) (CDCl_3_, 400 MHz).


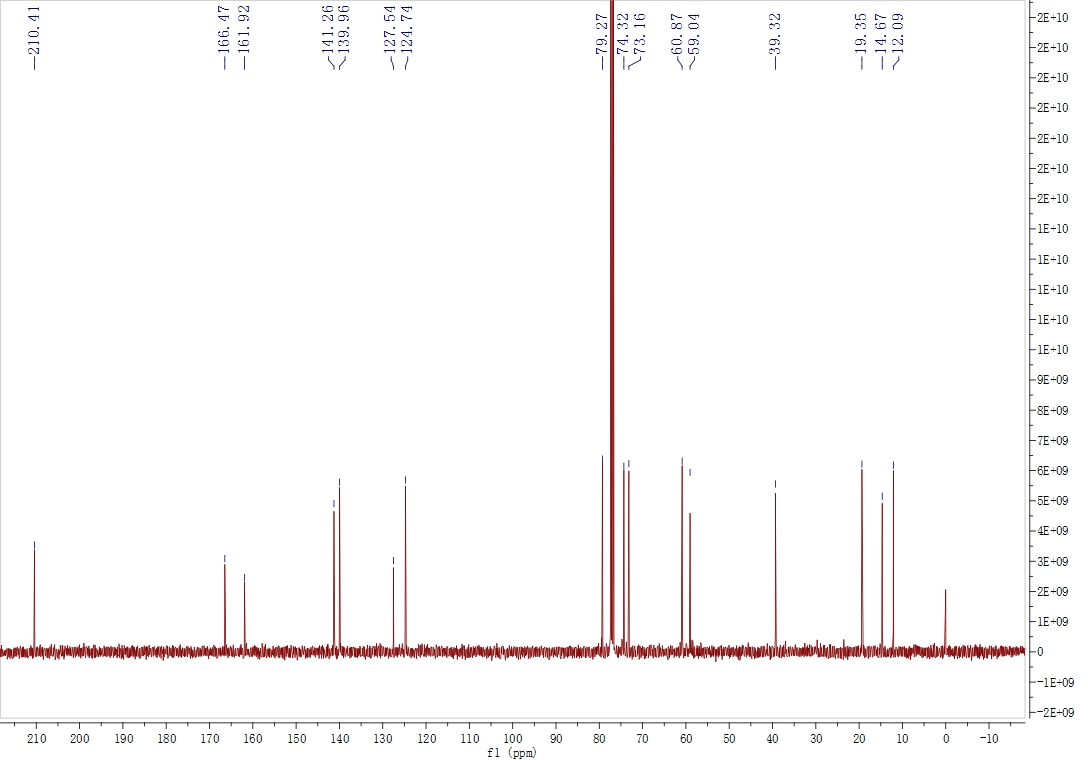


**Figure S7.** The ^13^C NMR spectrum of phomopsolide G (**1**) (CDCl_3_, 101 MHz).


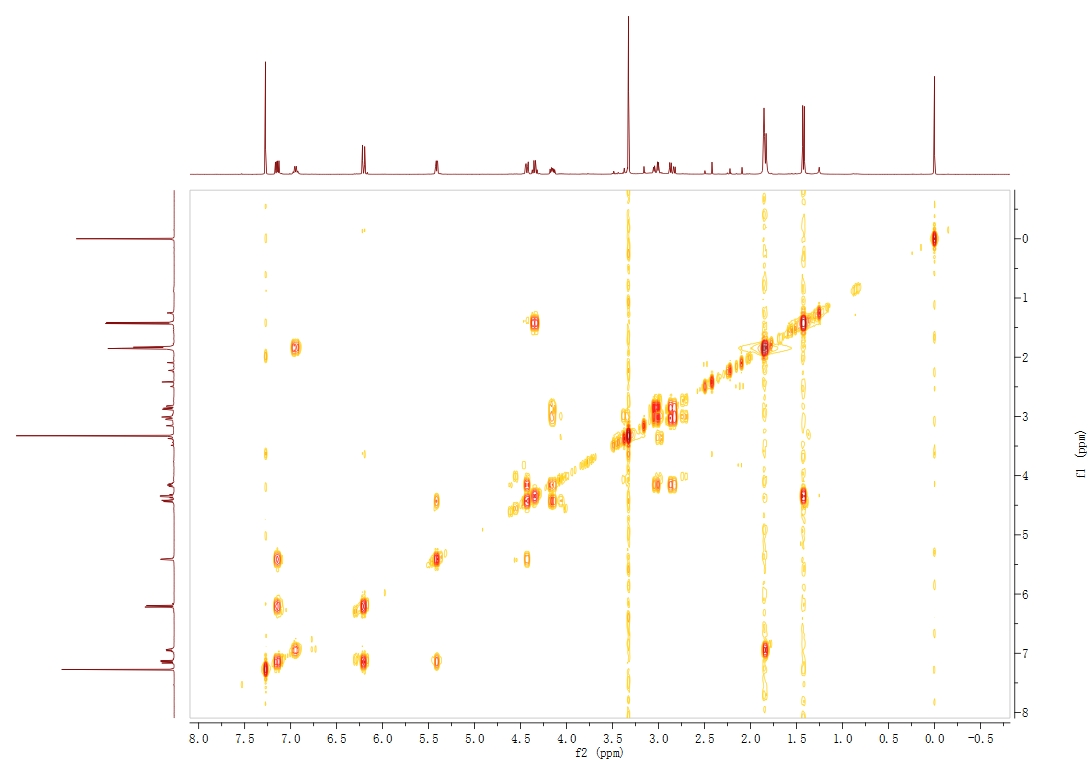


**Figure S8.** The ^1^H–^1^H COSY spectrum of phomopsolide G (**1**) (CDCl_3_, 400 MHz).

**
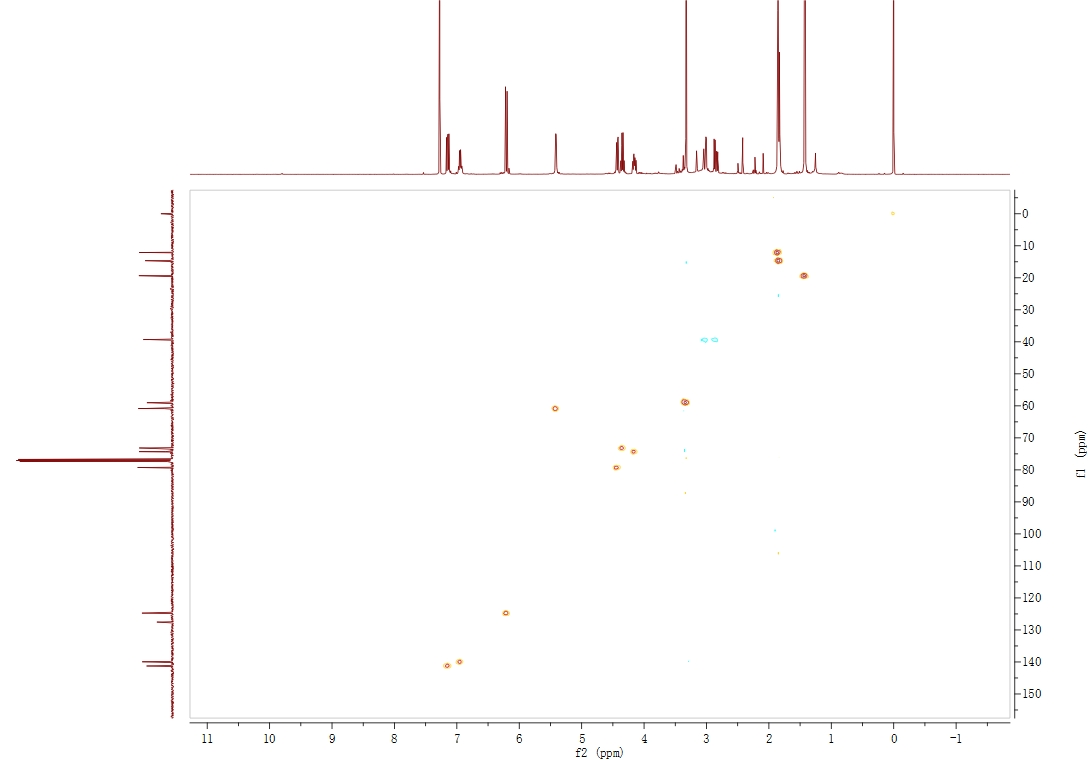
Figure S9.** The HSQC spectrum of phomopsolide G (**1**) (CDCl_3_, 400 MHz).


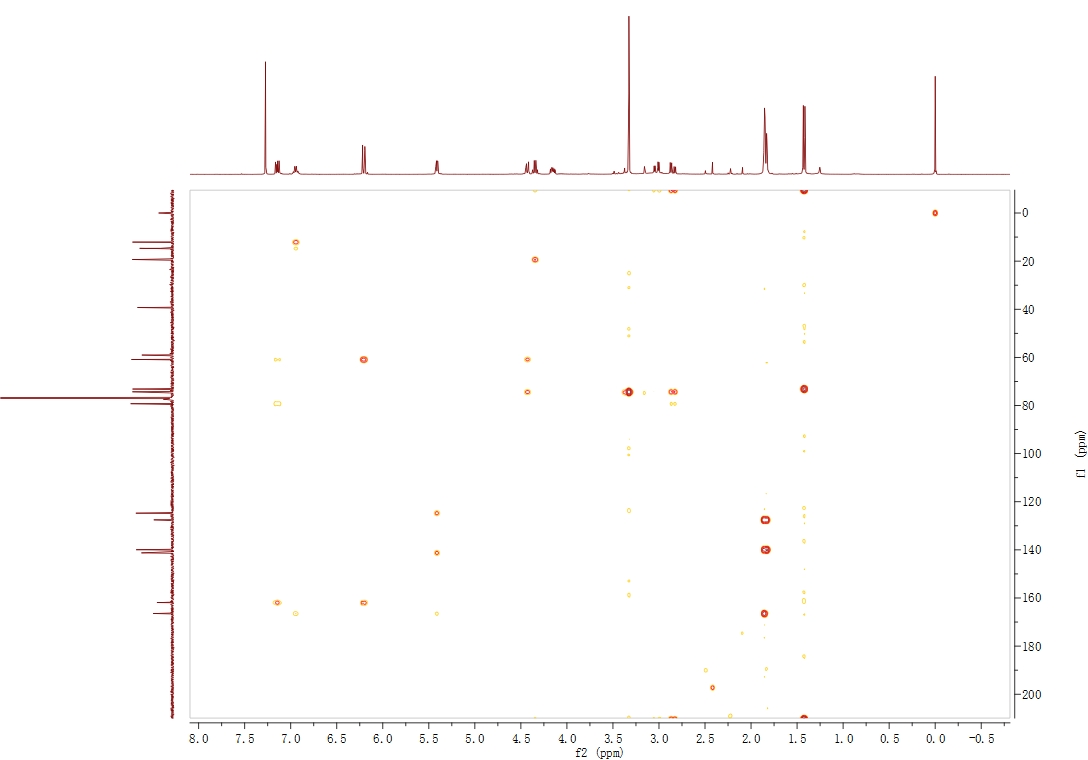


**Figure S10.** The HMBC spectrum of phomopsolide G (**1**) (CDCl_3_, 400 MHz).


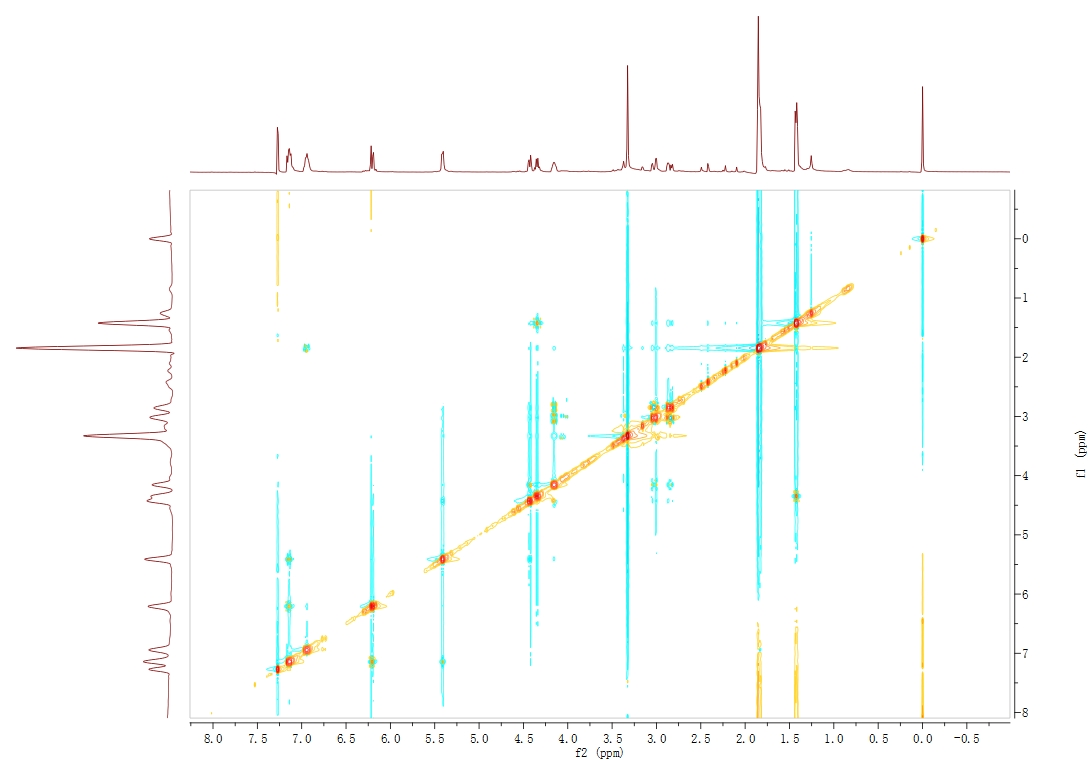


**Figure S11.** The NOESY spectrum of phomopsolide G (**1**) (CDCl_3_, 400 MHz).

**Figure S12.** The HR-MS of phomopsolide G (**1**).

**Figure S13.** The ^1^H NMR spectrum of phomopsolide F (**2**) (CDCl_3_, 400 MHz).

**Figure S14.** The ^13^C NMR spectrum of phomopsolide F (**2**) (CDCl_3_, 101 MHz).

**Figure S15.** The ^1^H NMR spectrum of phomopsolide B (**3**) (CDCl_3_, 400 MHz).

**Figure S16.** The ^13^C NMR spectrum of phomopsolide B (**3**) (CDCl_3_, 101 MHz).

**Figure S17.** The ^1^H NMR spectrum of phomopsolide A (**4**) (CDCl_3_, 400 MHz).

**Figure S18.** The ^13^C NMR spectrum of phomopsolide A (**4**) (CDCl_3_, 101 MHz).

**Figure S19.** The ^1^H NMR spectrum of (S,E)-6-(4-hydroxy-3-oxopent-1-en-1-yl)-2H-pyran-2-one (**5**) (CDCl_3_, 400 MHz).

**Figure S20.** The ^13^C NMR spectrum of (S,E)-6-(4-hydroxy-3-oxopent-1-en-1-yl)-2H-pyran-2-one (**5**) (CDCl_3_, 101 MHz).

**Figure S21.** The ^1^H NMR spectrum of catenioblin A (**6**) (Acetone-*d*_6_, 400 MHz).


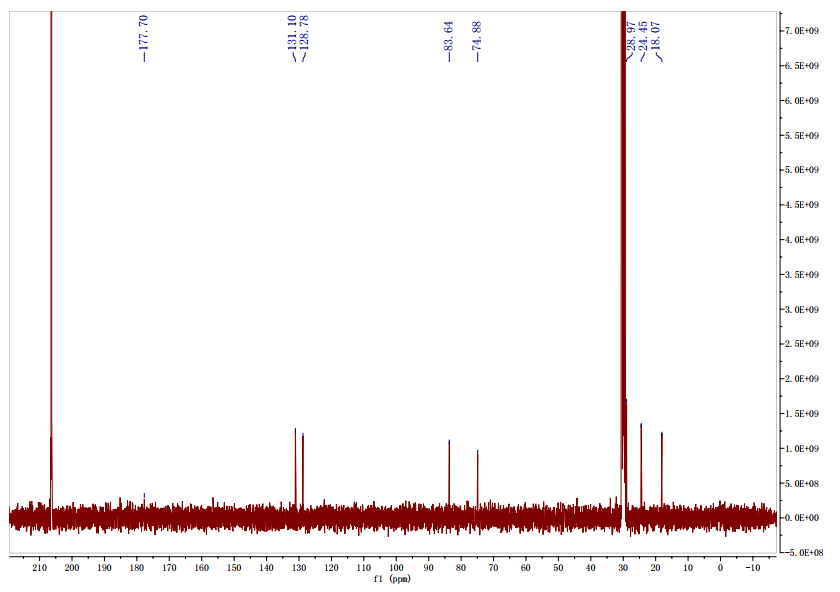


**Figure S22.** The ^13^C NMR spectrum of catenioblin A (**6**) (Acetone-*d*_6_, 101 MHz).


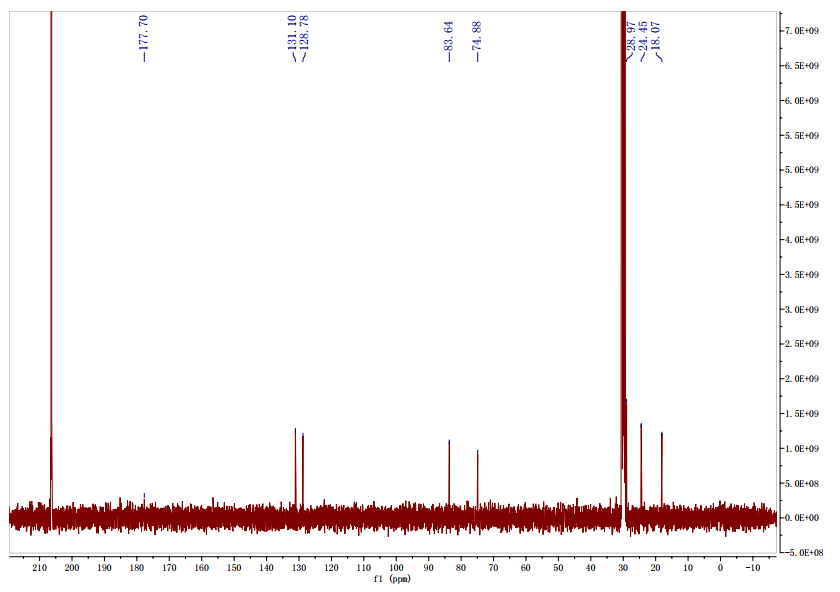


**Figure S23.** The ^1^H NMR spectrum of 2-minaline (**7**) (Acetone-*d*_6_, 400 MHz).

**Figure S24.** The ^13^C NMR spectrum of 2-minaline A (**7**) (Acetone-*d*_6_, 101 MHz).

**Figure S25.** The ^1^H NMR spectrum of 2-minaline (**8**) (Acetone-*d*_6_, 400 MHz).

**Figure S26.** The ^13^C NMR spectrum of 2-minaline A (**8**) (Acetone-*d*_6_, 101 MHz).

**TABLE S1** Cytotoxicity of compounds **1~5** and doxorubicin hydrochloride.

| Compounds | IC_50_ (μM) | | |
| --- | --- | --- | --- |
|  | HepG2 | A549 | MDA-MB-231 |
| **1** | 89.91±8.35 | 107.65±0.74 | 53.97±2.98 |
| **2** | 183.92±6.74 | 178.90±9.92 | 128.65±17.51 |
| **3** | 36.71±4.22 | 81.77±9.32 | 26.21±0.76 |
| **4** | 83.81±4.97 | 36.54±9.20 | 23.06±2.51 |
| **5** | 30.11±3.26 | 84.51±1.35 | 64.34±8.58 |
| Doxorubicin hydrochloride | 0.2±0.03 | 0.2±0.09 | 0.5±0.03 |

The results were expressed as mean ± standard deviation.
